# Individual Predisposition, Household Clustering and Risk Factors for Human Infection with *Ascaris lumbricoides*: New Epidemiological Insights

**DOI:** 10.1371/journal.pntd.0001047

**Published:** 2011-04-26

**Authors:** Martin Walker, Andrew Hall, María-Gloria Basáñez

**Affiliations:** 1 Department of Infectious Disease Epidemiology, School of Public Health, Imperial College London, St Mary's Campus, London, United Kingdom; 2 Centre for Public Health Nutrition, School of Life Sciences, University of Westminster, London, United Kingdom; The George Washington University Medical Center, United States of America

## Abstract

**Background:**

Much of our current understanding of the epidemiology of *Ascaris lumbricoides* infections in humans has been acquired by analyzing worm count data. These data are collected by treating infected individuals with anthelmintics so that worms are expelled intact from the gastrointestinal tract. Analysis of such data established that individuals are predisposed to infection with few or many worms and members of the same household tend to harbor similar numbers of worms. These effects, known respectively as individual predisposition and household clustering, are considered characteristic of the epidemiology of ascariasis. The mechanisms behind these phenomena, however, remain unclear. In particular, the impact of heterogeneous individual exposures to infectious stages has not been thoroughly explored.

**Methodology/Principal Findings:**

Bayesian methods were used to fit a three-level hierarchical statistical model to *A. lumbricoides* worm counts derived from a three-round chemo-expulsion study carried out in Dhaka, Bangladesh. The effects of individual predisposition, household clustering and household covariates of the numbers of worms per host (worm burden) were considered simultaneously. Individual predisposition was found to be of limited epidemiological significance once household clustering had been accounted for. The degree of intra-household variability among worm burdens was found to be reduced by approximately 58% when household covariates were included in the model. Covariates relating to decreased affluence and quality of housing construction were associated with a statistically significant increase in worm burden.

**Conclusions/Significance:**

Heterogeneities in the exposure of individuals to infectious eggs have an important role in the epidemiology of *A. lumbricoides* infection. The household covariates identified as being associated with worm burden provide valuable insights into the source of these heterogeneities although above all emphasize and reiterate that infection with *A. lumbricoides* is inextricably associated with acute poverty.

## Author Summary

Numerous analyses have found that people infected with roundworm (*Ascaris lumbricoides*) are predisposed to harbor either many or few worms. Members of the same household also tend to harbor similar numbers of worms. These phenomena are called individual predisposition and household clustering respectively. In this article, we use Bayesian methods to fit a statistical model to worm count data collected from a cohort of participants at baseline and after two rounds of re-infection following curative treatment. We show that individual predisposition is extremely weak once the clustering effect of the household has been accounted for. This suggests that predisposition is of limited importance to the epidemiology of roundworm infection. Further, we show that over half of the variability in average worm counts among households is explained by household risk factors. This implies that exposures to infectious roundworm eggs shared by household members are important determinants of household clustering. We argue that these results support the hypothesis proposed in the literature that the household is a key focus of roundworm transmission.

## Introduction

Much of our understanding of the epidemiology of *Ascaris lumbricoides* infections of humans has been acquired from the analysis of worm counts collected from infected individuals. The only practical way of obtaining such data is by chemo-expulsion. This procedure is best performed using anthelmintic drugs which paralyse gut-dwelling worms [1] so that they are expelled intact in the feces. The number of *A. lumbricoides* per host (worm burden) is the most important epidemiological variable with respect to the parasite's transmission potential and population dynamical behavior [2], as well as to the degree of individual and community morbidity [3].

Worm counts have been used to explore a number of aspects of the epidemiology of *A. lumbricoides* infection at both individual and household levels. At the individual level, “predisposition” describes the observed association between an individual's worm burden recovered after treatment, with the worm burden recovered after a period of re-infection and subsequent treatment. This phenomenon has been demonstrated frequently between two consecutive estimates of worm burden [4], [5], [6], [7], [8] and also over multiple rounds of treatment [9], [10], [11]. Predisposition is also evident at the household level: worm burdens tend to be associated among members of the same household [12], [13], [14] and average household worm burdens tend to be similar between rounds of treatment and re-infection [7], [15]. (For a review of predisposition to soil-transmitted helminthiases see Keymer and Pagel [16] and to *A. lumbricoides* in particular see Holland [17].)

The causes of predisposition at both the individual and household level are incompletely understood. Heterogeneities in exposure, innate (genetic) and immunologically-mediated susceptibility are likely to contribute [16], [17], [18]. While advances have been made in immunoepidemiology [19], [20] and genetically mediated susceptibility[21], [22], [23], little progress has been made in understanding the role of exposure to infective stages. This is largely due to the practical difficulties in measuring exposure [24]; estimation has been restricted to the measurement of concentrations of fecal silica as a proxy for soil contamination of food and geophagic activity [25], [26], [27]. Patterns of exposure may be inferred indirectly by exploring risk factors for worm burden. Numerous studies have identified factors associated with high *A. lumbricoides* egg output (those published since 2004 are described by Scott [28]), but only three have used worm counts as the dependent variable [14], [29], [30]. These studies have identified household-, agricultural-, host sex- and poverty-related factors associated with *A. lumbricoides* worm burdens.

The majority of chemo-expulsion studies were carried out between the early 1970s and the late 1980s (Table S1). Since this time, many statistical approaches have become increasingly accessible to parasitologists and easier to implement as research tools with personal computers. Such approaches include generalized linear models (GLMs) for non-normally distributed errors [31], [32], and longitudinal or hierarchical (random effects) models for repeated measures or clustered data [33], [34]. Bayesian methods provide a unifying framework with which to handle these and increasingly complex models [34], [35], affording a powerful tool to the analyze epidemiological and parasitological data [36], [37]. Many of these statistical methods have not before been applied to data on worm counts. For example, household clustering has largely been explored by dichotomising individual worm burdens as either “heavy” or “light” using an arbitrary threshold and estimating whether the number of worms per household observed, and the number expected by chance, are statistically significantly different [7]. This is a useful hypothesis-testing approach but does not quantify the clustering effect of interest [38]. Dichotomisation of continuous data also incurs a loss of statistical power. Hierarchical modelling is a more powerful and suitable approach which is becoming increasingly used for quantifying, and accounting for, the effects of household clustering in other helminth infections of humans [39], [40], [41], [42].

In this study, we explore evidence for individual predisposition, household clustering, and household risk factors for worm burdens of *A. lumbricoides* by analysing data from the largest of the chemo-expulsion studies conducted to date (Table S1). We define a statistical model capable of quantifying the effects of multiple, and potentially interacting, epidemiological phenomena by exploiting the longitudinal (multiple measurements made on a cohort) and hierarchical (individuals within households) structure of the data. Specifically, we examine the following: a) the interplay between individual predisposition and household clustering; b) the extent to which clustering of infections within households is explained by socioeconomic, physical and cultural differences among households, and c) the relative risk of worm burdens associated with these household variables.

## Methods

### Study Area and Data Collection

Data were collected in Mirpur, an urban suburb of Dhaka, Bangladesh between 1988 and 1989 by Hall and colleagues [11]. Briefly, households were visited by these authors and all their occupants invited to take part in the study with the aim of recruiting as many individuals as possible. Each participating household was administered a basic questionnaire to describe socio-economic status and household characteristics. These variables are listed in Table S2. A dose of pyrantel pamoate was given to each consenting subject and their stools were collected for a period of 48 hours post-treatment. The worms recovered (*A. lumbricoides*) from the feces of each individual were sexed and counted. Treatments and worm counts were repeated on two further occasions at six-monthly intervals. Pyrantel pamoate paralyzes *A. lumbricoides* in the gut so they are expelled intact from the gut by peristalsis [1] with a “cure” rate of approximately 88% [43]. Hence, these data provide a reliable and accurate measure of the number of worms (male and female) per host. The population of worms recovered after the first round of chemotherapy is termed the “baseline” population, after the second round of chemotherapy, the “first re-infection” population, and after the third and final round, the “second re-infection” population.

### Ethics Statement

This paper is concerned only with analyzing data previously collected by Hall and colleagues [11]. All analyses were conducted using anonymized data. In the original data collection study, informed consent was obtained in the following manner. A written statement was read to either the mother or father (usually the mother) of all children in the same household that were taking part in the study. The statement explained the aim of the study, what was to happen, telling them that they could refuse to take part or drop out at any time, and asking if they were willing to take part. The form was left with the household if they wanted to take advice from either religious or community leaders or if the father was absent and the mother wanted to defer to him to decide. One person, usually the father, signed or applied their thumb print (if the subject could not write) for all people in the household. This, however, did not mean that everyone in the household was able to participate. For example, there were relatively fewer adolescent and adult male participants because they tended to be out at work during the day and so could not collect their stools, which was a voluntary process.

Approval was given by the Ethical Review Committee of the International Centre for Diarrhoeal Disease Research, Dhaka, Bangladesh.

### Sample Size and Data Structure

**Figure 1:**
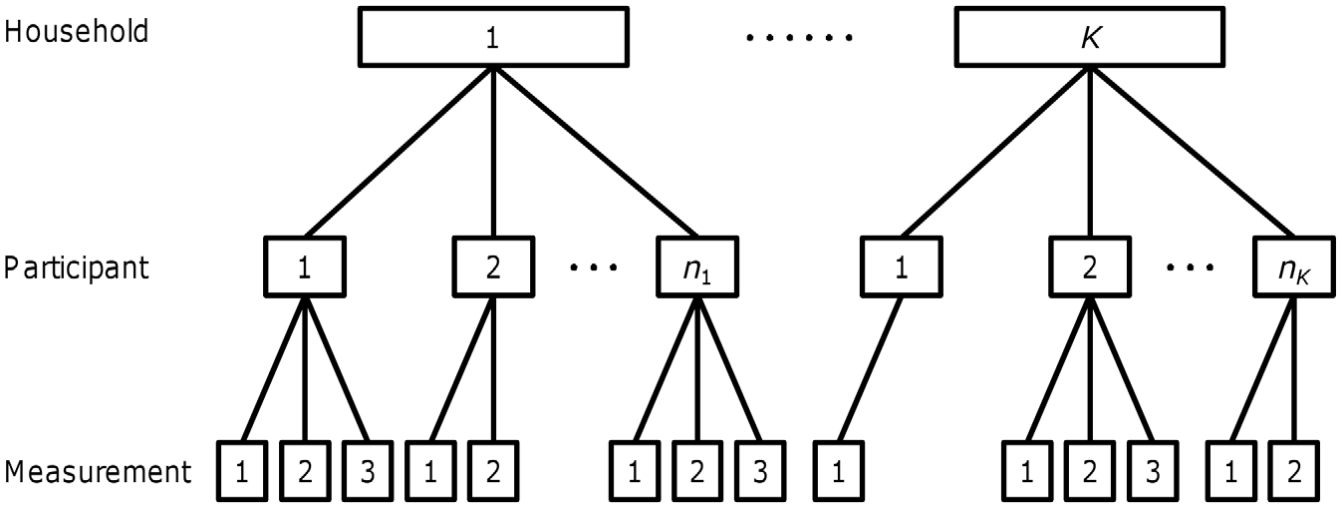
An illustration of the hierarchical structure of the data on *Ascaris lumbricoides* worm counts. Participants live in households such that *n_k_* participants live in household *k* and there are *K* households all together (*K = *459, see main text “*Sample Size, Data Structure and Missing Values*”). The total number of participants is 1,795. Each participant contributes at most three measurements of worm burden, one after each round of chemo-expulsive treatment with pyrantel pamoate, and at least one measurement (after the first round of treatment). Participants who were not “satisfactorily de-wormed” (see main text “*Sample Size, Data Structure and Missing Values*”) at a given round of treatment were not subsequently followed up.

A total of 2,929 subjects from 502 households originally enrolled to participate in the study. Participants were excluded from the study for any one of the following reasons: if stools were not collected for at least 48 h after treatment or if subjects reported not collecting all their stools; if a subject returned no worms although *A. lumbricoides* eggs had been seen in the fecal sample examined before treatment; or if a subject returned only male worms but eggs had been seen in the fecal sample collected before treatment. The subjects not excluded according to these criteria were classified as having been de-wormed satisfactorily [44]. Participants who were not satisfactorily de-wormed at a given round of treatment were not subsequently followed up. On this basis, 1,765 participants from 459 households were satisfactorily de-wormed after the first round of treatment, 1,257 after the first six-month period of re-infection and 1,017 after the second re-infection period (Table 1). Overall, a maximum of three worm burdens were measured from each subject (one at baseline and a further two after consecutive six month periods of re-infection). Figure 1 illustrates the hierarchical and longitudinal structure of the data. All data analyzed were anonymized at the individual level retaining characteristics such as age, sex, ethnicity and household, but not allowing personal identification.

**Table 1:** The number of participants who were “satisfactorily de-wormed” after each round of chemo-expulsive treatment.

Round of treatment*	Participants satisfactorily de-wormed	Participants excluded	Households sampled	Households excluded
Baseline	1,765	1,164	459	43
First re-infection	1,257	508	364	105
Second re-infection	1,017	240	321	43

*Baseline, first and second re-infection populations refer to the first, second and third treatments respectively.

### Overview of Statistical Model

Here we give a brief description of the key features of the statistical model which was fitted to the data. A formal definition can be found in Text S1. The model is structured into three nested hierarchies; multiple measurements per individual and measurements made on multiple members of the same household (Figure 1). The correlations between potentially dependent measures are accounted for by two random effects, the variances of which are denoted 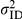 and 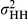. The subscripts ID and HH and the parameters to which they pertain quantify the magnitude of individual predisposition and household-level clustering respectively.

Informed by a previous analysis of these data [44], worm burdens were assumed to be negatively binomially distributed. Covariates were included at each hierarchical level. At the measurement-level (measurements made on a single participant), the population from which the worm burden was measured was the sole covariate (i.e. baseline, first or second re-infection population). The individual-level covariates included host age, defined as an 11-level categorical variable using the groupings defined in Hall et al. [11], [44], and sex. In addition, age-population and age-sex interactions were included. Adjustments for host age were necessary because, in this population, baseline worm burden varies with age in a “convex” manner [11], [44] typical of *A. lumbricoides* infection [2]. The sex-age interaction was incorporated because analyses by Hall et al. [44] had suggested that adult women tend to harbor higher worm burdens than adult men, with no apparent difference between the sexes in children. The host age-population interaction allowed the rate of re-infection to vary with age, where the former is defined as the proportion of the baseline worm burden attained after 6 months. Two previous studies have demonstrated age dependency in the rate of re-infection, showing that children become re-infected at a faster rate than adults [4], [5].

At the household-level, additive covariates were included pertaining to the socioeconomic status of the household, the quality of construction of the house and the hygiene facilities available to household members. An interaction between the ethnicity of the household and whether rent was paid was also included. This was done because of the pronounced differences in circumstances between Biharis and Bangladeshis. Biharis live effectively as refugees and tend to be confined to an extremely crowded and poverty-stricken refugee camp. Those not confined to the camp were more likely to pay rent for their home. By contrast, the difference in living conditions between households paying or not paying rent in the Bangladeshi community was much less conspicuous. The average worm burden in each population stratified by each household covariate can be found in Table S2.

### Reduction of Household Covariates

Preliminary analyses were carried out in order to reduce the number of household covariates (Table S2), eliminating those that did not contribute enough to the likelihood of the fitted model to warrant inclusion in the subsequent analysis. This was achieved using an Akaike's information criterion (AIC)-based [45] forward and backward stepwise selection procedure implemented using the stepAIC function in R [46], [47]. For this procedure, the simplest model was defined as that described in the above section *Summary of Statistical Model*, minus the household-level covariates and the random effects. The most complex model included the household covariates listed in Table S2, but again omitted the random effects. By removing random effects in this way, the potential correlations among infection intensities at the individual- and household-levels are ignored. This is a conservative approach to the preliminary elimination of explanatory variables because variables will contribute relatively more to the likelihood of the fitted model when the variability arising from the clustering of data at each hierarchical level (random effects) is ignored. This technique has been used previously for the reduction of covariates in a hierarchical statistical model of *Ascaris suum* infections in swine [48]. The number of worms per host was assumed to be negatively binomially distributed with an unknown overdispersion parameter (estimated from the model). Models were fitted by maximum likelihood using the glm.nb function in R [46]. The most parsimonious model arrived at by the selection procedure included household ethnicity, number of sleepers, number of children, number of rooms, rent, floor type, source of water for washing dishes and latrine facility (Table 2; see Table S3 for coefficient estimates).

**Table 2:** The most parsimonious model of the worm burden of *Ascaris lumbricoides* ignoring random effects.

	DF†	Change in deviance‡	Residual DF†	Residual deviance	*p*-value
Null	NA	NA	4,038	5,704	NA
Age group	10	181.3	4,028	5,522	< 0.001
Population	2	132.0	4,026	5,390	< 0.001
Sex	1	28.8	4,025	5,361	< 0.001
Ethnicity of household	2	236.4	4,023	5,125	< 0.001
Rent paid per household	1	38.0	4,022	5,087	< 0.001
No. sleepers per household	2	12.3	4,020	5,075	0.002
No. children per household	3	19.4	4,017	5,055	< 0.001
Rooms per household	1	15.5	4,016	5,040	< 0.001
Floor construction of house	1	74.6	4,015	4,965	< 0.001
Household source of water for washing plates	3	18.6	4,012	4,947	< 0.001
Household latrine facility	2	22.8	4,010	4,924	< 0.001
Age group × Population	20	126.3	3,990	4,798	< 0.001
Age group × Sex	10	27.2	3,980	4,770	0.002
Ethnicity of household × Rent paid per household	2	20.1	3,978	4,750	< 0.001

**†:** Degrees of freedom.

**‡:** Deviance is the difference between a fitted model's log-likelihood and the maximum achievable log-likelihood.

### Model Fitting

The “full” model described in *Summary of Statistical Model* and nested models were fitted to the data using hierarchical Bayesian techniques in the Windows program for Bayesian inference using Gibbs Sampling (WinBUGS) [49]. Parameters were assigned non-informative priors [35], e.g., a normal distribution with mean = 0 and a variance = 1000 or, for the precision (1/variance) of random effects, a gamma distribution with shape and scale parameters = 0.001. Following techniques suggested by Gelman and Rubin [50] three starting values for the Gibbs sampling algorithm were assigned in order to asses convergence on the parameter posterior distributions and to check that our conclusions were not sensitive to the choice of starting values. In general, the first 20,000 samples of each chain were discarded as “burn in” and a further 40,000 samples were used to compute the posterior distributions. The goodness-of-fit/parsimony of each model was assessed using the Deviance Information criterion (DIC) [51]. This is a Bayesian generalization of AIC, based on a trade-off between the fit of the model to the data and its complexity. Like AIC, the smaller a model's DIC, the more parsimonious the fit.

## Results

A total of 8 models were fitted to the data, each incorporating a different combination of epidemiological features nested within the full model (Model 1 in Table 3). The full model included individual predisposition, household clustering and household covariates. The simplest or null model (Model 8 in Table 3) omitted these components. All models included the other features described in the Methods section entitled Summary of Statistical Model.

**Table 3:** Summary of the 8 models fitted to data on *Ascaris lumbricoides* worm counts.

Model	Epidemiological features	DIC*	Individual predisposition, 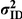 (95% BCI†)	Household clustering, 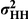 (95% BCI†)
1	Individual predisposition, household clustering and household covariates	27,761	0.097 (0.043, 0.19)	0.52 (0.37, 0.73)
2	Individual predisposition and household clustering	27,832	0.080 (0.027,0.15)	1.24 (0.86,1.80)
3	Individual predisposition and household covariates	27,985	0.67 (0.48, 0.93)	ND‡
4	Household clustering and household covariates	27,811	ND	0.55 (0.40, 0.77)
5	Individual predisposition only	28,208	1.17 (0.82, 1.64)	ND
6	Household covariates only	28,403	ND	ND
7	Household clustering only	27,878	ND	1.26 (0.88, 1.82)
8	None	28,718	ND	ND

*Deviance information criterion. A lower DIC indicates a more parsimonious fit.

**†:** Bayesian credible interval.

**‡:** Not defined.

The “best-fit” model according to the DIC is the full model (Table 3). Estimates of 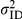 and 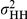 from each of the fitted models are also given in Table 3. Two aspects of these parameter estimates are noteworthy. First, the magnitude of household clustering is much larger when estimated from models that do not incorporate household covariates compared to estimates from models which do account for household covariates (comparing 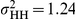 in Model 2 with 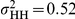 in Model 1 or 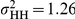 in Model 7 with 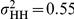 in Model 4, Table 3). Household clustering is reduced by approximately 58% having adjusted for household covariates. The second notable point is that the magnitude of individual predisposition is extremely small, except in models in which household clustering is unaccounted for (compare Model 3 or Model 5 vs. Model 1 or Model 2 in Table 3).

**Figure 2:**
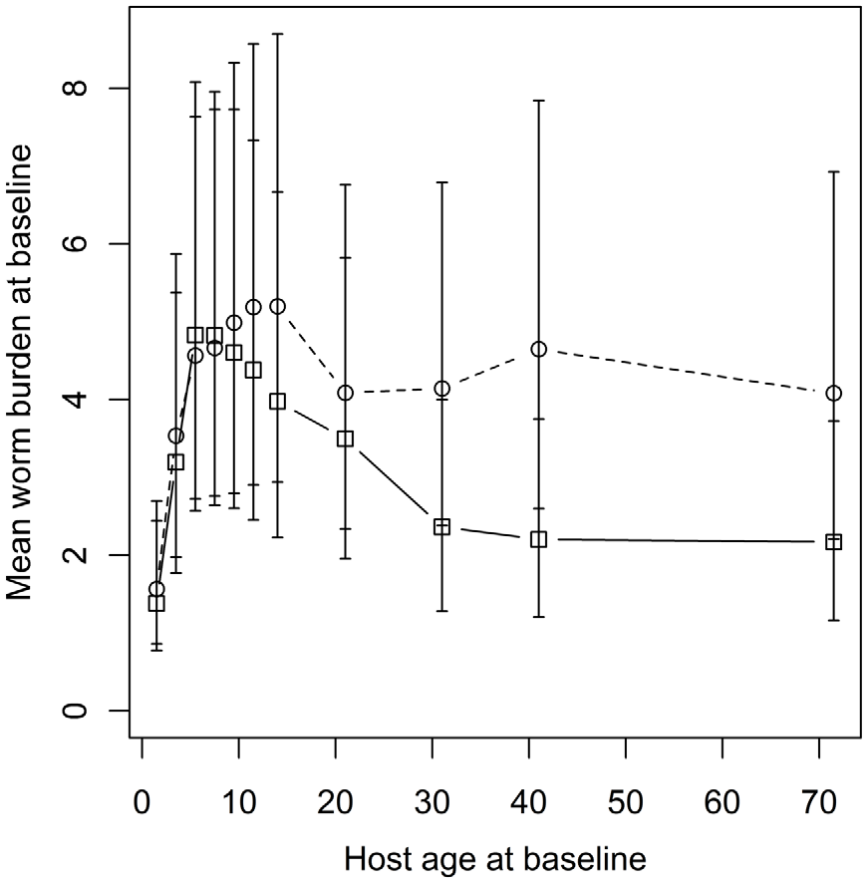
Mean worm burden at baseline vs. host age and sex. Fitted points are posterior means calculated across all households from the “full” model (Model 1, see Table 3). Squares and solid lines denote males, circles and dashed lines females. Household risk factors are adjusted to their null levels, i.e. a Bangladeshi family of 2–4 sleeping members with no children, paying no rent, living in a house with a single room, an earth floor and private well and latrine facilities (see Table 4). Error bars represent 95% Bayesian credible intervals.

**Figure 3:**
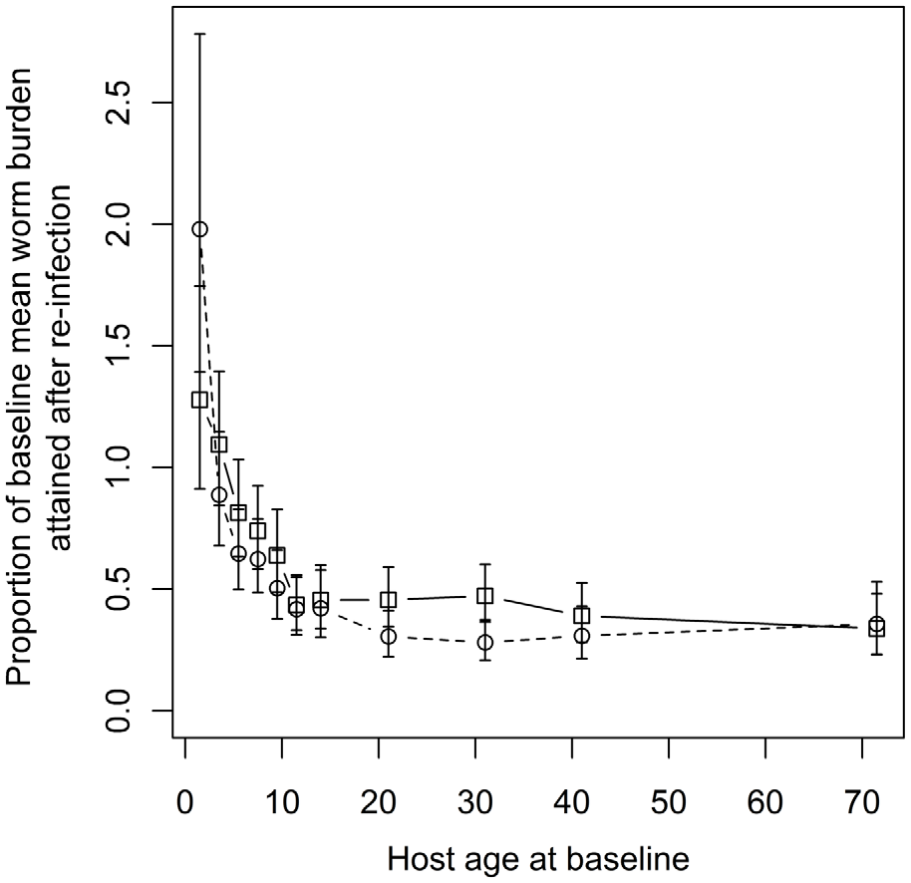
The proportion of the baseline mean worm burden after six months re-infection vs. host age. Fitted points are posterior means estimated from the “full” model (Model 1, see Table 3). Squares and solid lines denote the first re-infection population, circles and dashed lines the second re-infection population. Error bars represent 95% Bayesian credible intervals.

The fitted relationship between the mean worm burden at baseline, host age and sex estimated from Model 1 is depicted in Figure 2. This highlights the convex age-burden profile at baseline and the tendency of adult women to harbor heavier worm burdens than adult men. Also apparent are the wide 95% Bayesian credible intervals (BCI) which are, in part, the result of the additional uncertainty introduced by household clustering and, to a much lesser extent, individual predisposition. Figure 3 depicts the fitted relationship between the proportion of the baseline mean worm burden and host age in the first and second re-infection populations. The figure shows that children tend to re-acquire their pre-treatment worm burdens more rapidly than adults. Indeed children aged 1–4 years at baseline tended to re-acquire slightly heavier worm burdens in the first re-infection population than they had at baseline. Moreover, children aged 1–2 years at baseline had re-acquired twice their baseline worm burden in the second re-infection population. In contrast, teenagers and adults harbored approximately 50% of their baseline worm burden in both re-infection populations.

Table 4 gives the posterior means and 95% BCIs for the relative risks of household covariates on the worm burden of *A. lumbricoides* estimated from Model 1. The BCIs for the following variables do not include 1, indicating statistically significantly more intense infections in: Bihari households, households using a common tap to wash dishes, households with an earth floor, and those with no latrine.

**Table 4:** The estimated relative risks of the worm burden of *Ascaris lumbricoides* for household variables.

Variable	Category	Relative risk* (95% BCI†)
**No. sleepers per household**		
	2-4	1‡
	5-6	1.15 (0.91, 1.42)
	7+	1.04 (0.80, 1.33)
**No. children per household**		
	0	1‡
	1-2	1.10 (0.78, 1.51)
	3-4	1.19 (0.80, 1.67)
	5+	1.38 (0.89, 2.04)
**No. rooms per household**		
	1	1‡
	2+	0.88 (0.73, 1.06)
**Floor construction of house**		
	Cement	1‡
	Earth	1.44 (1.20, 1.70)
**Household source of water for washing plates**		
	Own well/tube well	1‡
	Common well/tube well	1.31 (0.87, 1.92)
	Own tap	1.33 (0.89, 1.91)
	Common tap	1.43 (1.01, 1.98)
**Household latrine facility**		
	Own latrine	1‡
	Shared latrine	1.04 (0.84, 1.26)
	None	1.32 (1.08, 1.59)
**Ethnicity of household x Rent paid per household**		
	Bangladeshi, no rent	1‡
	Bihari, no rent	1.97 (1.55, 2.33)
	Mixed, no rent	1.74 (1.00, 2.81)
	Bangladeshi, paying rent	0.76 (0.57, 1.00)
	Bihari, paying rent	1.44 (1.05, 1.92)
	Mixed, paying rent	0.50 (0.14,1.28)

*Estimates were derived from the fit of the “full” model (Model 1, see Table 3).

**†:** Bayesian credible interval.

**‡:** Baseline relative risk is by definition equal to 1.

## Discussion

The modeling approach taken in this paper to analyze data on *A. lumbricoides* worm counts has enabled the effects of multiple epidemiological phenomena and their interplay with one another to be considered into a single coherent inference framework for the first time in the study of human ascariasis. There are two key findings. First, the degree of individual predisposition to worm burden is extremely small once the clustering of infections within households has been accounted for. Second, approximately 58% of residual intra-household variability (clustering) is accounted for by household covariates, the effects of which have been quantified in the form of relative risks.

The limited impact of individual predisposition on the fitted models suggests that heterogeneity in susceptibility or exposure among members of the same household is of little epidemiological importance. This result complements that of Chan et al. [52] who failed to find any difference among the associations between the worm burdens of parents and their (genetically related) children and between unrelated parents. These authors surmised that any genetic basis to individual predisposition must be overwhelmed by household-related behavioral or environmental factors. The results of this study lend support to this supposition demonstrating that individual predisposition is weak [11], [16] and swamped by putative effects within the household [52].

The genetic component of susceptibility to *A. lumbricoides* infection [21], [22], [23] is not challenged by our results. Children within a household are presumably closely genetically related to each other and to their parents. Consanguineous relationships among parents were also fairly common in this community (12% of wives reported being directly related to their husbands). In this way, household members are often closely genetically related and so clustering may be partly due to shared genetics. However, the lower household clustering with the inclusion of household covariates suggests that household-related exposures also play an important role in transmission. Conway et al. [53] reached similar conclusions regarding the cause of clustering within households of the soil-transmitted helminth (STH) *Strongyloides stercoralis* by analyzing prevalence data (presence or absence of eggs in feces) also collected from the study described in this paper. These authors found that household clustering of *S. stercoralis* was only partially explained by household risk factors and surmised, “Household aggregation of *S. stercordis* may be partly due either to close contact person to person transmission within households, or to familial genetic predisposition to infection.”

The importance of the household in the transmission of the three most prevalent STH infections (*A. lumbricoides*, *Trichuris trichiura* and the hookworm species *Ancylostoma duodenale* and *Necator americanus* [54]) was first considered by Otto et al. in the 1930s [55]. In 1996, Cairncross et al [56] suggested that the household and public environments are fundamental “arenas for disease transmission” in formulating their “domain theory” of transmission. The view is that *A. lumbricoides* is primarily transmitted within the “domestic (household) domain”, a notion based on a variety of epidemiological observations, including clustering of infections within households [24]. Direct evidence for this assertion has, however, only recently been presented. Criscione et al [57] found that *A. lumbricoides* collected from a Nepalese community were genetically clustered within households and that nearby households shared genetically similar worms. These results are in accordance with the peri-domiciliary environment as the focus of transmission. This novel work also demonstrated the power of using the genetic information from individual worms to gain insight into the mechanisms behind observed epidemiological patterns at the host and household levels.

Many studies have used statistical models to explore putative risk factors for *A. lumbricoides* infection and other STHs. Surprisingly few, however, have employed estimates of worm burden as the dependent variable, often using weak, dichotomous data on presence or absence of worms (for recent examples see [40], [58]). In areas of moderate to high transmission where the average worm burden per host is high, prevalence is not a suitable response measure because individuals with a high exposure to infectious larvae will be indiscernible from those less exposed. This arises because of the non-linear relationship between infection prevalence and worm burden [2], [59]. Studies that have used either direct (worm counts) or indirect (egg counts) measures of worm burden have identified an array of behavioral, cultural, occupational, socio-economic and host sex-related risk factors (e.g. [14], [29], [30], [60]). The risk factors identified in the present study relate broadly to socio-economic status: individuals with large worm loads tend to live in households with an earth floor, without a latrine and rely on a common tap for their washing water. The average worm burden of Bihari and mixed ethnicity households not paying rent was approaching twice that of Bangladeshi households not paying rent (relative risk = 1.85 (1.49, 2.28), Table 4). In Bangladesh, Biharis are an impoverished minority group living as refugees in overcrowded insanitary camps, a legacy of the secession of East Pakistan in the creation of Bangladesh after the Bangladeshi War of Independence [61].The null component of all of the fitted models has two important features hitherto relatively unexplored in the literature on *A. lumbricoides*. These are: a) the interaction between age at baseline and the rate of re-infection, and b) the effect of host sex on worm burden. Figure 3 indicates that the rate of return to baseline worm burden is slower with increasing age. Indeed children aged 1–2 years at baseline reacquired, on average, a heavier worm burden after six months of re-infection in both the first and second re-infection populations compared to their worm burden at baseline. Anderson and May [2] identified that if the rate at which individuals acquire parasites remains constant then the rate of return to baseline/endemic worm burdens depends on the life-expectancy of the parasite. Assuming *A. lumbricoides* live for 1–2 years [2] one would expect hosts to re-acquire, on average, 40-60% of their baseline worm burden after six months of re-infection. This is what is seen for teenagers and adults (Figure 3). The higher proportion of the baseline worm burden attained by children suggests that the rate at which they are acquiring worms is increasing as the cohort ages over the one year study period. Similar differences between the relative rates of re-infection in children compared with adults have been reported in other longitudinal chemo-expulsion studies [4],[5],[9]. These authors cited age-specific rates of exposure as the likely cause. The results presented here are in accordance with this explanation. In adults, aging by a maximum of one year is unlikely to affect patterns of exposure. In children, exposure which is behaviorally mediated may change rapidly with age especially over the first three years of life as they learn to walk and explore their environment, which will increase their exposure to *A. lumbricoides* eggs.

The inclusion of host age and sex as interacting covariates indicated that adult women tend to have higher worm burdens than adult men, with no discernable difference between children by sex (Figure 2). The most forthcoming explanation is that exposure has a sex-specific component. This has also been demonstrated to be the case in other nematode infections of humans such as *Onchocerca volvulus* [62]. In the study community, teenage and adult males tend to spend their days at work away from the household. By contrast females seldom leave the peri-domiciliary environment. Similar sex differences have been reported between children in a Madagascan community where boys spend their days away from the village and girls remain at home looking after the younger children [30]. The consequence is that it was possible to de-worm satisfactorily more women than men. At baseline, 38% of the de-wormed participants over 16 years were male; this figure was 34% after each period of re-infection. By contrast, 49% of those under 16 were male. The sampling bias in favour of adult females was induced by the absence of men from the household during the day which made them less able to collect their feces. This is likely to have had two effects on our results. First, there would have been a slight loss of power in discerning between the worm burdens of adult men and women. Second, and more importantly, it is conceivable that those men not de-wormed harbored fewer worms that those who were de-wormed because they were away from the household (the focus of transmission) more often. If this was the case then over results would underestimate the true difference between the worm burdens of men and women. That is, men may have been infected with, on average, even fewer worms than the results suggest.

In the late 1980's, when the study described in this paper was being carried out, the first public-private partnership between Merck and the Onchocerciasis Control Programme was being forged to deliver donated ivermectin to treat onchocerciasis [63]. Numerous public-private initiatives have since ensued, fuelling a rise in mass drug administration- (MDA) based helminth control programs and heeding the calls of the World Health Organization's (WHO) campaign against neglected tropical diseases [64], [65]. The recommended protocol for MDA against the STHs is annual or biannual treatment with benzimidazole drugs (albendazole or mebendazole) targeted at school-age children, as children tend to harbor the highest burden of STHs and suffer the most from the insidious effects of chronic infection [66]. The schools infrastructure facilitates high coverage permitting cost-effectiveness treatment [67], [68], [69] and regular de-worming has beneficial effects on the nutrition, growth, physical fitness and cognitive performance of school-age children [54].

Despite the unequivocal benefits of school-based de-worming, it is inevitable that such an approach will miss potentially heavily infected groups outside of the target population. For instance, in the study population, the Bihari refugees were approximately twice as heavily infected as Bangladeshis (Table 4). In general, the epidemiological relevance of missing potentially heavily infected groups will be highly location-specific and will critically depend on the number of individuals comprising the overlooked groups and on the portion of the worm burden harbored by them. Identifying and targeting such groups (in addition to school-age children) prior to treatment would amount to a selective treatment strategy [70] albeit possibly at a household- rather than at an individual-level. Such an approach requires potentially costly prior epidemiological assessment and may not be as cost-effective [71], [72], although cost-effectiveness will be improved in areas of high population density such as the Bihari refugee camp described in this study. In locations where control efforts are successful in suppressing worm burdens, the relevance of consistently missing heavily infected groups will be increased and more community-specific strategies may be necessary to complement the school-based approach.In this analysis, we have exploited the flexibility of a Bayesian statistical modeling approach to simultaneously consider a number of epidemiological phenomena associated with *A lumbricoides* infections of humans. This approach has enabled for the first time exploration within the same framework of the interplay between individual predisposition, household clustering and household risk factors. We have found that the magnitude of individual predisposition to high or low worm burdens became extremely small once the effect of the household has been accounted for. That is, the predominant unit of predisposition is the household rather than the individual. Furthermore, a number of household risk factors associated with worm numbers have been identified which together account for approximately 58% of the variation in worm counts among households. These risk factors, like others identified before, are invariably associated with socio-economic status and relative affluence even in what is overall an extremely poor community. Thus, while highlighting the importance of heterogeneous exposures to transmission, such risk factors, above all, confirm that *A. lumbricoides* is associated with acute poverty, and that its control is inextricably linked to help achieving the Millennium Development Goals [73], [74].

## Supporting Information

Table S1 Summary of chemo-expulsion studies to have collected adult *Ascaris lumbricoides* from humans by study starting date
(0.08 MB DOC)

Table S2 Summary of household explanatory variables
(0.08 MB DOC)

Table S3 Coefficient estimates of the most parsimonious model arrived at by the preliminary selection procedure
(0.10 MB DOC)

Text S1 Definition of statistical model
(0.10 MB DOC)
