# Supplementary material for: Individual Predisposition, Household Clustering and Risk Factors for Human Infection with Ascaris lumbricoides: New Epidemiological Insights
Source: PLoS Negl Trop Dis. 2011 Apr 26;5(4):e1047. doi: 10.1371/journal.pntd.0001047 (PMC3082514; doi:10.1371/journal.pntd.0001047)
Supplement: Table S1 — Summary of chemo-expulsion studies to have collected adult Ascaris lumbricoides from humans by study starting date (0.08 MB DOC) [file pntd.0001047.s001.doc]

**Table S1. Summary of chemo-expulsion studies to have collected adult *Ascaris lumbricoides* from humans by study starting date.**

| Location | Study period | Number of participants^*^ | Age of participants (years) | Drug | Study type | Reference |
| --- | --- | --- | --- | --- | --- | --- |
| Iran | 1972-1973 | 652 | ≤ 5-40+ | pyrantel pamoate | cross-sectional | [1] |
| Iran | 1973-1974 | 252 | 0-45+ | pyrantel pamoate | cross-sectional | [2] |
| South Korea | 1975-1978 | 853 | NA^†^ | pyrantel pamoate | cross-sectional | [3] |
| South Korea | 1977-1980 | 31-78^†^ | NA^†^ | pyrantel pamoate | cross-sectional | [4,5] |
| Bangladesh | 1982 | 203 | 0.5-15 | pyrantel pamoate | cross-sectional | [6] |
| Burma | 1982 | 239 | ≤ 5-39+ | levamisole | cross-sectional | [7] |
| Philippines | 1983 | 308 | primary school | flubendazole | cross-sectional | [8] |
| Panama | 1983-1984 | 203 | 3-5 | levamisole | cross-sectional | [9] |
| India | 1984-1985 | 224 | ≤ 2-65+ | pyrantel pamoate | longitudinal | [10] |
| Philippines | 1985-1986 | 150 | 0-14 | pyrantel pamoate | cross-sectional | [11] |
| Mexico | 1986 | 118 | 2-10 | mebendazole | longitudinal | [12] |
| Nigeria | 1987 | 808 | 5-16 | levamisole | longitudinal | [13] |
| St Lucia | NA^†^ | 113 | 1-30+ | mebendazole | longitudinal | [14] |
| Bangladesh^‡^ | 1988-1989 | 1765 | 1-98 | pyrantel pamoate | longitudinal | [15] |
| Madagascar | 1990-1992 | 428 | 5-11 | pyrantel pamoate | cross-sectional | [16] |
| China | 1994-1995 | 222 | 0-55+ | pyrantel pamoate | cross-sectional | [17] |
| Nepal | 1998-2003 | 1,007 | 3-85 | albendazole | longitudinal | [18] |

* The number of participants from whom the worm burden was successfully assessed after anthelmintic treatment. For longitudinal studies this refers to the number after the first round of treatment.

† Not available in the published paper.

‡ The study from which the data used in the current paper were derived.

**Table S1 References**

1. Arfaa F, Ghadirian E (1977) Epidemiology and mass-treatment of ascariasis in six rural communities in central Iran. American Journal of Tropical Medicine and Hygiene 26: 866-871.

2. Croll NA, Anderson RM, Gyorkos TW, Ghadirian E (1982) The population biology and control of *Ascaris lumbricoides* in a rural community in Iran. Transactions of the Royal Society of Tropical Medicine and Hygiene 76: 187-197.

3. Seo B-S, Cho S-Y, Chai J-Y (1979) Frequency distribution of *Ascaris lumbricoides* rural koreans with special reference on the effect of changing endemicity. Korean Journal of Parasitology 18: 105-112.

4. Seo B-S, Chai J-Y (1980) Comparative efficacy of various interval mass treatment on *Ascaris lumbricoides* infection in Korea. Korean Journal of Parasitology 18: 145-151.

5. Seo B-S, Chai J-Y (1980) Effect of two-month interval mass chemotherapy on the reinfection of *Ascaris lumbricoides* in Korea. Korean Journal of Parasitology 18: 153-163.

6. Martin J, Keymer A, Isherwood RJ, Wainwright SM (1983) The prevalence and intensity of *Ascaris lumbricoides* infections in Moslem children from northern Bangladesh. Transactions of the Royal Society of Tropical Medicine and Hygiene 77: 702-706.

7. Thein-Hlaing, Than-Saw, Htay-Htay-Aye, Myint-Lwin, Thein-Maung-Myint (1984) Epidemiology and transmission dynamics of *Ascaris lumbricoides* in Okpo village, rural Burma. Transactions of the Royal Society of Tropical Medicine and Hygiene 78: 497-504.

8. Cabrera BD (1984) Reinfection and infection rates of ascariasis in relation to seasonal variation in the Philippines. The Southeast Asian Journal of Tropical Medicine and Public Health 15: 394-401.

9. Holland CV, Crompton DW, Taren DL, Nesheim MC, Sanjur D, et al. (1987) *Ascaris lumbricoides* infection in pre-school children from Chiriqui Province, Panama. Parasitology 95: 615-622.

10. Elkins DB, Haswell-Elkins M, Anderson RM (1986) The epidemiology and control of intestinal helminths in the Pulicat Lake region of Southern India. I. Study design and pre- and post-treatment observations on *Ascaris lumbricoides* infection. Transactions of the Royal Society of Tropical Medicine and Hygiene 80: 774-792.

11. Monzon RB, Cabrera BD, Cruz AC, Baltazar JC (1990) The "crowding effect" phenomenon in *Ascaris* *lumbricoides*. Southeast Asian Journal of Tropical Medicine and Public Health 21: 580-585.

12. Forrester JE, Scott ME, Bundy DAP, Golden MHN (1988) Clustering of *Ascaris lumbricoides* and *Trichuris trichiura* infections within households. Transactions of the Royal Society of Tropical Medicine and Hygiene 82: 282-288.

13. Holland CV, Asaolu SO, Crompton DWT, Stoddart RC, Macdonald R, et al. (1989) The epidemiology of *Ascaris lumbricoides* and other soil-transmitted helminths in primary school children from Ile-Ife, Nigeria. Parasitology 99: 275-285.

14. Bundy DA, Cooper ES, Thompson DE, Didier JM, Simmons I (1987) Epidemiology and population dynamics of *Ascaris lumbricoides* and *Trichuris trichiura* infection in the same community. Transactions of the Royal Society of Tropical Medicine and Hygiene 81: 987-993.

15. Hall A, Anwar KS, Tomkins AM (1992) Intensity of reinfection with *Ascaris lumbricoides* and its implications for parasite control. Lancet 339: 1253-1257.

16. Kightlinger LK, Seed JR, Kightlinger MB (1995) The epidemiology of *Ascaris lumbricoides*, *Trichuris trichiura*, and hookworm in children in the Ranomafana rainforest, Madagascar. Journal of Parasitology 81: 159-169.

17. Peng W, Zhou X, Cui X (2002) Comparison of the structures of natural and re-established populations of *Ascaris* in humans in a rural community of Jiangxi, China. Parasitology 124: 641-647.

18. Williams-Blangero S, Subedi J, Upadhayay RP, Manral DB, Rai DR, et al. (1999) Genetic analysis of susceptibility to infection with *Ascaris lumbricoides*. American Journal of Tropical Medicine and Hygiene 60: 921-926.
