# Supplementary material for: Individual Predisposition, Household Clustering and Risk Factors for Human Infection with Ascaris lumbricoides: New Epidemiological Insights
Source: PLoS Negl Trop Dis. 2011 Apr 26;5(4):e1047. doi: 10.1371/journal.pntd.0001047 (PMC3082514; doi:10.1371/journal.pntd.0001047)
Supplement: Table S2 — Summary of household explanatory variables (0.08 MB DOC) [file pntd.0001047.s002.doc]

**Table S2. Summary of household explanatory variables.**

| Variable and category (per household) | No. households | Individuals sampled at baseline | Mean worm burden (standard error) | | |
| --- | --- | --- | --- | --- | --- |
|  |  |  | Baseline population | First re-infection population | Second re-infection population |
| Ethnicity × Rent |  |  |  |  |  |
| Bangladeshi, no rent | 60 | 245 | 11.22 (0.88) | 8.07 (0.95) | 6.22 (0.76) |
| Bihari, no rent | 277 | 1104 | 20.12 (0.64) | 14.70 (0.60) | 12.78 (0.60) |
| Mixed, no rent | 9 | 43 | 22.53 (6.56) | 14.70 (2.65) | 10.93 (2.38) |
| Bangladeshi, paying rent | 68 | 212 | 8.21 (0.82) | 4.30 (0.54) | 4.22 (0.79) |
| Bihari, paying rent | 42 | 154 | 12.06 (1.23) | 10.05 (1.93) | 11.96 (2.11) |
| Mixed, paying rent | 3 | 7 | 0.86 (0.34) | 0.5 (0.5) | NA |
| No. sleepers | | | | | |
| 2-4 | 129 | 294 | 12.99 (0.99) | 9.28 (1.05) | 8.05 (1.02) |
| 5-6 | 176 | 663 | 16.72 (0.76) | 12.20 (0.75) | 10.80 (0.80) |
| 7+ | 154 | 808 | 18.10 (0.73) | 13.61 (0.70) | 11.92 (0.65) |
| No. children | | | | | |
| 0 | 29 | 71 | 16.17 (2.37) | 6.27 (1.55) | 3.17 (1.04) |
| 1-2 | 180 | 552 | 13.94 (0.72) | 9.02 (0.74) | 8.66 (0.81) |
| 3-4 | 181 | 755 | 17.61 (0.78) | 13.64 (0.75) | 11.36 (0.74) |
| 5+ | 69 | 387 | 19.10 (1.01) | 15.49 (0.97) | 13.99 (0.90) |
| No. children aged 2-5 | | | | | |
| 0 | 172 | 577 | 15.73 (0.70) | 9.44 (0.73) | 8.27 (0.78) |
| 1 | 191 | 734 | 16.80 (0.80) | 12.87 (0.79) | 11.06 (0.67) |
| 2-3 | 96 | 454 | 17.89 (0.95) | 15.21 (0.86) | 13.91 (0.98) |
| No. rooms |  |  |  |  |  |
| 1 | 362 | 1362 | 16.86 (0.53) | 12.98 (0.53) | 11.77 (0.52) |
| 2+ | 97 | 403 | 16.32 (1.04) | 10.48 (0.96) | 8.18 (0.98) |
| Floor |  |  |  |  |  |
| Earth | 321 | 1244 | 18.98 (0.59) | 14.55 (0.57) | 12.44 (0.56) |
| Cement | 138 | 521 | 11.36 (0.69) | 6.92 (0.71) | 7.01 (0.72) |
| Roof^†^ |  |  |  |  |  |
| Bamboo / plastic / card | 105 | 407 | 19.06 (1.03) | 12.34 (0.87) | 11.52 (0.81) |
| Tin | 313 | 1213 | 16.80 (0.57) | 13.08 (0.58) | 11.51 (0.59) |
| Cement | 41 | 145 | 9.63 (1.31) | 6.97 (1.60) | 4.78 (0.92) |
| Source of drinking water^‡^ | | | | | |
| Own well / tube well | 7 | 31 | 10.94 (1.85) | 6.10 (1.89) | 6.40 (2.38) |
| Common well / tube well | 34 | 154 | 17.87 (1.48) | 11.97 (1.30) | 10.32 (1.13) |
| Own tap | 73 | 273 | 12.66 (1.04) | 12.71 (1.48) | 9.74 (1.16) |
| Common tap | 345 | 1307 | 17.59 (0.57) | 12.57 (0.52) | 11.37 (0.55) |
| Source of water for washing plates^‡^ | | | | | |
| Own well / tube well | 19 | 74 | 11.82 (1.19) | 9.45 (1.81) | 6.52 (1.78) |
| Common well / tube well | 41 | 173 | 18.14 (1.43) | 12.27 (1.22) | 11.81 (1.18) |
| Own tap | 72 | 268 | 12.13 (1.01) | 12.72 (1.51) | 9.74 (1.18) |
| Common tap | 327 | 1250 | 17.82 (0.59) | 12.55 (0.54) | 11.30 (0.56) |
| Latrine facility^§^ | | | | | |
| Own latrine | 130 | 530 | 14.27 (0.75) | 10.29 (0.77) | 8.78 (0.68) |
| Shared latrine | 140 | 498 | 13.74 (0.86) | 8.85 (0.74) | 8.46 (0.78) |
| None | 189 | 737 | 20.52 (0.79) | 16.05 (0.78) | 13.80 (0.78) |
| Income^*^(Taka per month) | | | | | |
| 0-800 | 133 | 510 | 17.47 (0.91) | 12.51 (0.79) | 11.44 (0.88) |
| 801-1000 | 99 | 340 | 17.37 (1.08) | 13.97 (1.14) | 10.83 (0.93) |
| 1001-1600 | 113 | 492 | 18.44 (0.92) | 12.67 (0.88) | 12.14 (0.93) |
| 1600+ | 114 | 423 | 13.34 (0.85) | 10.79 (1.00) | 9.00 (0.87) |

† A single household had a plastic / card roof which was amalgamated into the bamboo group

‡ Tube wells and wells were differentiated in the raw data, here they are amalgamated

§ The latrine facilities of each household were inferred from the usual place of mother’s defecation

* Stratified into quartiles
