# Supplementary material for: Individual Predisposition, Household Clustering and Risk Factors for Human Infection with Ascaris lumbricoides: New Epidemiological Insights
Source: PLoS Negl Trop Dis. 2011 Apr 26;5(4):e1047. doi: 10.1371/journal.pntd.0001047 (PMC3082514; doi:10.1371/journal.pntd.0001047)
Supplement: Table S3 — Coefficient estimates of the most parsimonious model arrived at by the preliminary selection procedure (0.10 MB DOC) [file pntd.0001047.s003.doc]

**Table S3. Coefficient estimates of the most parsimonious model arrived at by the preliminary selection procedure**

| **Variable** | **Category** | **Coefficient (standard error)** | ***p-*value**^†^ |
| --- | --- | --- | --- |
| **Age group x Population** | | | |
|  | 1-2, baseline (intercept) | 0.93 (0.21) | < 0.001 |
|  | 3-4,baseline | 0.71 (0.17) | < 0.001 |
|  | 5-6,baseline | 0.99 (0.17) | < 0.001 |
|  | 7-8,baseline | 0.97 (0.17) | < 0.001 |
|  | 9-10,baseline | 0.93 (0.18) | < 0.001 |
|  | 11-12,baseline | 0.90 (0.18) | < 0.001 |
|  | 13-16,baseline | 0.97 (0.19) | < 0.001 |
|  | 17-26,baseline | 0.67 (0.18) | < 0.001 |
|  | 27-36,baseline | 0.39 (0.19) | 0.036 |
|  | 37-46,baseline | 0.25 (0.19) | 0.2 |
|  | 47+,baseline | 0.32 (0.20) | 0.11 |
|  | 1-2, first re-infection | 0.18 (0.17) | 0.29 |
|  | 3-4, first re-infection | -0.053 (0.22) | 0.81 |
|  | 5-6, first re-infection | -0.34 (0.22) | 0.12 |
|  | 7-8, first re-infection | -0.37 (0.21) | 0.087 |
|  | 9-10, first re-infection | -0.53 (0.23) | 0.019 |
|  | 11-12, first re-infection | -0.99 (0.23) | < 0.001 |
|  | 13-16, first re-infection | -1.03 (0.23) | < 0.001 |
|  | 17-26, first re-infection | -0.91 (0.22) | < 0.001 |
|  | 27-36, first re-infection | -0.90 (0.22) | < 0.001 |
|  | 37-46, first re-infection | -0.96 (0.24) | < 0.001 |
|  | 47+, first re-infection | -1.14 (0.26) | < 0.001 |
|  | 1-2, second re-infection | 0.61 (0.18) | < 0.001 |
|  | 3-4, second re-infection | -0.67 (0.23) | 0.0034 |
|  | 5-6, second re-infection | -1.02 (0.23) | < 0.001 |
|  | 7-8, second re-infection | -1.02 (0.22) | < 0.001 |
|  | 9-10, second re-infection | -1.17 (0.24) | < 0.001 |
|  | 11-12, second re-infection | -1.39 (0.24) | < 0.001 |
|  | 13-16, second re-infection | -1.49 (0.25) | < 0.001 |
|  | 17-26, second re-infection | -1.61 (0.24) | < 0.001 |
|  | 27-36, second re-infection | -1.74 (0.24) | < 0.001 |
|  | 37-46, second re-infection | -1.73 (0.26) | < 0.001 |
|  | 47+, second re-infection | -1.65 (0.28) | < 0.001 |
| **Age group x Sex** | | | |
|  | 1-2, female | 0.024 (0.15) | 0.87 |
|  | 3-4, female | -0.01 (0.19) | 0.97 |
|  | 5-6, female | -0.14 (0.18) | 0.45 |
|  | 7-8, female | 0.06 (0.18) | 0.73 |
|  | 9-10, female | 0.05 (0.19) | 0.79 |
|  | 11-12, female | 0.21 (0.19) | 0.28 |
|  | 13-16, female | 0.17 (0.20) | 0.4 |
|  | 17-26, female | 0.23 (0.20) | 0.24 |
|  | 27-36, female | 0.50 (0.20) | 0.012 |
|  | 37-46, female | 0.62 (0.21) | 0.0028 |
|  | 47+, female | 0.43 (0.23) | 0.056 |
| **No. sleepers per household** | | | |
|  | 2-4 | 0 | NA |
|  | 5-6 | 0.18 (0.07) | 0.0084 |
|  | 7+ | 0.11 (0.080) | 0.16 |
| **No. children per household** | | | |
|  | 0 | 0 | NA |
|  | 1-2 | 0.14 (0.12) | 0.25 |
|  | 3-4 | 0.23 (0.13) | 0.075 |
|  | 5+ | 0.29 (0.14) | 0.038 |
| **No. rooms per household** | | | |
|  | 1 | 0 | NA |
|  | 2+ | -0.08 (0.05) | 0.12 |
| **Floor construction of house** | | | |
|  | Earth | 0 | NA |
|  | Cement | -0.41 (0.05) | < 0.001 |
| **Household source of water for washing plates** | | | |
|  | Own well / tube well | 0 | NA |
|  | Common well / tube well | 0.31 (0.12) | 0.0084 |
|  | Own tap | 0.33 (0.11) | 0.0035 |
|  | Common tap | 0.39 (0.10) | < 0.001 |
| **Household latrine facility** | | | |
|  | Own latrine | 0 | NA |
|  | Shared latrine | -0.02 (0.06) | 0.7 |
|  | None | 0.17 (0.05) | < 0.001 |
| **Ethnicity of household x Rent paid per household** | | | |
|  | Bangladeshi, no rent | 0 | NA |
|  | Bangladeshi, paying rent | -0.21 (0.08) | 0.012 |
|  | Bihari, no rent | 0.54 (0.06) | < 0.001 |
|  | Bihari, paying rent | 0.20 (0.11) | 0.073 |
|  | Mixed, no rent | 0.49 (0.13) | <0.001 |
|  | Mixed, paying rent | -2.53 (0.56) | <0.001 |
| **Overdispersion parameter** | | 0.66 (0.016) | NA^‡^ |

* For household risk factors the baseline is given with coefficient = 0 and *p*-value = NA (not applicable) for improved interpretability of the estimated coefficients

† Calculated using a *z*-score which assumes the sampling distribution of the coefficient is normal

‡ Not applicable, the overdispersion parameter is by definition always > 0
