# Supplementary material for: Individual Predisposition, Household Clustering and Risk Factors for Human Infection with Ascaris lumbricoides: New Epidemiological Insights
Source: PLoS Negl Trop Dis. 2011 Apr 26;5(4):e1047. doi: 10.1371/journal.pntd.0001047 (PMC3082514; doi:10.1371/journal.pntd.0001047)
Supplement: Text S1 — Definition of statistical model (0.10 MB DOC) [file pntd.0001047.s004.doc]

**Text S1**

*Definition of Statistical Model*

At the measurement level (first hierarchical level), we assumed that the *i*^th^ worm burden measured from host *j* living in household *k*, $y_{ijk}$, was a realisation of a negative binomially distributed (NBD) random variable with mean $\mu_{ijk}$and overdispersion parameter ,

(1)

Following log-linear modelling techniques [1,2], the natural logarithm of $\mu_{ijk}$ was adjusted for population, as denoted by the indicator variables $x_{1ijk}$for the first-, and $x_{2ijk}$ for the second, re-infection population,

(2)

Here $\varepsilon_{jk}$ is a random effect allowing for a correlation among worm burdens measured on individual *j*, accounting for predisposition. On the natural logarithmic scale (Equation (2)), the distribution of $\varepsilon_{jk}$ was assumed to be normal with mean 0 and variance $\delta_{\text{ID}}^{2}$. Consequently on the scale of the response, $\exp\left(\varepsilon_{jk}\right)$ is log-normally distributed with variance, $\sigma_{\text{ID}}^{2}=\exp\left(2\delta_{\text{ID}}^{2}\right)-\exp\left(\delta_{\text{ID}}^{2}\right)$. This variance term is a quantitative measure of individual-predisposition.

The parameters , and in Equation (2) denote, respectively, the natural logarithms of the baseline worm burden and the proportions of the baseline worm burden in the first and second re-infection populations. These were adjusted for individual-level covariates so as to capture the trends reported in the literature and from previous analyses of these data (see main text *Overview of Statistical Model*). Age-dependent rates of re-infection [3,4,5] were captured by adjusting and $\alpha_{2j}$ in 10 age groups (1-2, 3-4, 5-6, 7-8, 9-10, 11-12, 13-16, 17-26, 27-36, 37-46, 47+, where 1-2 is baseline) denoted by the indicator variables $x_{1jk},x_{2jk},...,x_{10jk}$,

$\alpha_{1j}=\beta_{1}+\beta_{2}x_{1jk}+,...,+\beta_{11}x_{10jk},$ (3)

$\alpha_{2j}=\beta_{12}+\beta_{13}x_{1jk}+,...,+\beta_{22}x_{10jk}.$ (4)

The age and gender dependency of the baseline worm burden [6] was captured by modelling as dependent on an interaction between age group and gender. To this end, a further 11 indicator variables were defined in addition to those indicative of age group. Together these covariates permitted differential adjustment for males and females in each age group,

$\alpha_{0jk}=\beta_{0k}+\beta_{23}x_{1jk}+\beta_{24}x_{2jk}+,...,+\beta_{43}x_{21jk}+\varepsilon_{k}.$ (5)

Here $\varepsilon_{k}$ denotes the second random effect which accounts for the potential correlation among worm burdens measured from members of the same household. As with the previous random effect term, $\exp\left(\varepsilon_{k}\right)$ is log-normally distributed on the response scale with variance, $\sigma_{\text{HH}}^{2}=\exp\left(2\delta_{\text{HH}}^{2}\right)-\exp\left(\delta_{\text{HH}}^{2}\right)$, which quantifies the degree of household clustering.

The household-level covariates (risk factors) remaining statistically significant after preliminary reduction (see *Reduction of Household-Level Variables* and Table 2 in the main text) were incorporated into the model by adjusting the intercept term $\beta_{0k}$ for the indicator variables $x_{1k}+x_{2k}+,...,+x_{15k}$,

$\beta_{0k}=\gamma_{0}+\gamma_{1}x_{1k}+\gamma_{2}x_{2k}+,...,+\gamma_{15}x_{15k}.$ (6)

The relative risk of a given household covariate was calculated from the estimated coefficients in Equation (6). For example, if $x_{1k}=1$ denotes a Bihari household not paying rent then the corresponding relative risk of infection intensity is $\exp\left(\gamma_{1}\right)$.

**References**

1. McCullagh P, Nelder JA (1989) Generalized Linear Models London: Chapman & Hall.

2. Booth JG, Casella G, Friedl H, Hobert JP (2003) Negative binomial loglinear mixed models. Statistical Modelling 3: 179-191.

3. Elkins DB, Haswell-Elkins M, Anderson RM (1986) The epidemiology and control of intestinal helminths in the Pulicat Lake region of Southern India. I. Study design and pre- and post-treatment observations on *Ascaris lumbricoides* infection. Transactions of the Royal Society of Tropical Medicine and Hygiene 80: 774-792.

4. Thein-Hlaing, Myint-Lwin (1987) Reinfection of people with *Ascaris lumbricoides* following single, 6-month and 12-month interval mass chemotherapy in Okpo village, rural Burma. Transactions of the Royal Society of Tropical Medicine and Hygiene 81: 140-146.

5. Hall A, Anwar KS, Tomkins AM (1992) Intensity of reinfection with *Ascaris lumbricoides* and its implications for parasite control. Lancet 339: 1253-1257.

6. Hall A, Anwar KS, Tomkins A (1999) The distribution of *Ascaris lumbricoides* in human hosts: a study of 1765 people in Bangladesh. Transactions of the Royal Society of Tropical Medicine and Hygiene 93: 503-510.
